# Comparison of superior airway dimensions and cephalometric anatomic landmarks between 8–12-year-old children with obstructive sleep apnea and healthy children using CBCT images

**DOI:** 10.34172/joddd.2022.003

**Published:** 2022-05-29

**Authors:** Farzad Emsaeili, Amirhouman Sadrhaghighi, Mahnaz Sadeghi-Shabestari, Parastou Nastarin, Aliakbar Niknafs

**Affiliations:** ^1^Department of Oral and Maxillofacial Radiology, Faculty of Dentistry, Tabriz University of Medical Sciences, Tabriz, Iran; ^2^Department of Orthodontics, Faculty of Dentistry, Tabriz University of Medical Sciences, Tabriz, Iran; ^3^Immunology Research Center, TB and lung research center, Children hospital, Tabriz University of Medical Sciences, Tabriz, Iran; ^4^Dentist, Private Practice, Tabriz, Iran

**Keywords:** Airway obstruction, Cone-beam computed tomography, Obstructive sleep apnea

## Abstract

**Background.** The etiology of obstructive sleep apnea (OSA) syndrome in children significantly differs from adults. In previous studies, only some of the indices have been investigated using CBCT. This study compares all the measurable indices of airway dimensions and anatomical cephalometric landmarks between children with OSA and healthy ones using cone-beam computed tomography (CBCT).

**Methods.** Dimensions of the airway and cephalometric values were measured on CBCT scans of 50 children aged 8–12 (25 patients with OSA and 25 healthy subjects) and then compared between the two groups. The results of this study were analyzed with independent *t* test using SPSS 17 at a significance level of *P*<0.05.

**Results.** Area, length, volume, anteroposterior length, and size of the upper airway in subjects with OSA were lower than those in healthy children, while the average values of SNA, SNB, and ANB in the OSA group were higher than those in the healthy group (*P*=0.366, *P*=0.012, and *P*=0.114, respectively). Also, BaSN, PNS/AD1, and PNS/AD2 measurements in subjects with OSA were lower than healthy subjects (*P*=0.041, *P*=0.913, and *P*=0.015, respectively). In addition, the width and anteroposterior length of the upper airway, SNB, BaSN, PNS/AD1, and PNS/AD2 indices were significantly different between the healthy group and those with OSA (*P*<0.05).

**Conclusion.** Reduced upper airway dimensions, adenoid tissue enlargement, and cranial base flexion might play an important role in OSA development in children. However, most skeletal variables, such as the anteroposterior relationship of jaws and jaw rotation, were not significantly different between the two groups.

## Introduction

 Total or partial obstruction of the airway during sleep, called obstructive sleep apnea (OSA)/hypopnea,^1^ is a common disorder that affects 0.7%–2% of the pediatric population.^1,2^ It is usually determined by apnea-hypopnea index of > 5 events/hour, which is the number of total or partial obstruction events per hour during sleep.^3^ Major signs and symptoms of OSA include snoring, restless sleep, neurocognitive and behavioral problems, headaches, attention deficit, and hyperactivity.^2,4,5^ Some authors have suggested that if OSA is not treated during childhood, it might lead to drug and alcohol abuse in adult life.^6^

 The etiology of OSA in children is different from adults. In adults, ASO is usually accompanied by obesity, but most children with OSA are not obese. In contrast, adenotonsillar hypertrophy is considered one of the major causes of OSA in children.^7^ Some other causes of OSA in children include mandibular deficiency or retrusion, increased fat accumulation in the pharyngeal area, and neuromotor abnormalities.^8-10^ Also, it seems that children with OSA have a narrower airway than normal children.^10^

 The gold standard of OSA diagnosis is in-lab polysomnography which is expensive and time-consuming.^8^ Therefore, different tools have been designed for OSA screening, such as the Berlin questionnaire and STOP and STOP-Bang questionnaires that are completed in a few minutes and are valid.^11,12^

 There is a relationship between sleep disorders and children’s physical, emotional, and neurocognitive problems; therefore, OSA diagnosis is important in the childhood period.^4,13^ Studies have revealed that besides narrow upper airway (UA), aging, Berlin questionnaire high scores, and gender (male > female) are among the risk factors of OSA.^1^ Also, soft tissue to craniofacial space ratio, which is the ratio of upper airway soft tissue volume to nasopharyngeal and oropharyngeal craniofacial space, is higher in OSA patients.^14^ Some studies have used lateral cephalograms to evaluate UA dimensions in children with OSA, revealing decreased pharyngeal diameters at levels of the uvula, adenoids, and tongue,^15^ with a significant, positive correlation with MRI findings. Therefore, it is a valid method for retropharyngeal and nasopharyngeal measurements.^16^ Several studies have evaluated airways in OSA children using cone-beam computed tomography (CBCT) as a three-dimensional (3D) and precise imaging method, concluding that the presence and severity of OSA are correlated with lateral obstruction of UA and a narrow nasopharyngeal area.^1,17^

 This study aimed to compare superior airway dimensions and cephalometric anatomic landmarks between 8–12-year-old children with OSA and healthy children using CBCT images, which is the first study in this age group to the best of our knowledge. This study also helps determine specific measurements that suggest OSA possibility in children, leading to more precise diagnosis and effective treatment approaches.

## Methods

 This descriptive study was carried out on CBCT scans of 50 children aged 8–12 (25 patients with OSA and 25 healthy subjects), referring to the Department of Oral and Maxillofacial Radiology, Faculty of Dentistry, Tabriz University of Medical Sciences for different reasons.

 CBCTs of children in this age range were found in the department archives, and Berlin questionnaires were filled for every sample in the study using telephone calls.

 The inclusion criteria consisted of OSA confirmed by the Berlin questionnaire for the OSA group and scans of healthy children matched to those with OSA regarding gender and age from the archives, whose health was confirmed by the Berlin questionnaire as the control group. Exclusion criteria consisted of the presence of any syndromes or clefts, a history of surgery in the palatopharyngeal area, significant medical and growth conditions, a history of the face and neck trauma, upper airway anomalies, asthma, and any upper airway acute or chronic infection.^15^

 All the CBCT scans were obtained by a NewTom VGi cone-beam CT unit (Verona/Italy), and image reconstruction was conducted by NNT Viewer software (version 2.21). The images were observed on a 19-inch LCD monitor (190B Phillips, Netherlands) with a resolution of 1024*1208 and 32 bits by an OMF radiologist.

 The images were exported as DICOM (.dcm) files and then imported to Mimics10.10 (Materialise NV,Belgium) software for airway analysis.

 To determine the volumetric region of interest, a mid-sagittal picture was exported, and upper and lower limits of the UA region were defined as below:

*Upper limit:* A line parallel to the FH (Frankfurt Plane) at the level of the most distal point of the hard palate

*Lower limit:* A line parallel to the FH at the level of the most anterior-inferior point of the second cervical vertebra (C_2_).

 To evaluate the width and anteroposterior dimension of UA, an axial picture of the region with the smallest dimension was selected.

 UA length was measured from PNS to the most anterior-inferior point of C_2._

 Using Mimic software, the volume and cross-section area of UA were automatically measured in mm^3^ and mm^2^ in the oropharyngeal region.

**Figure 1:**
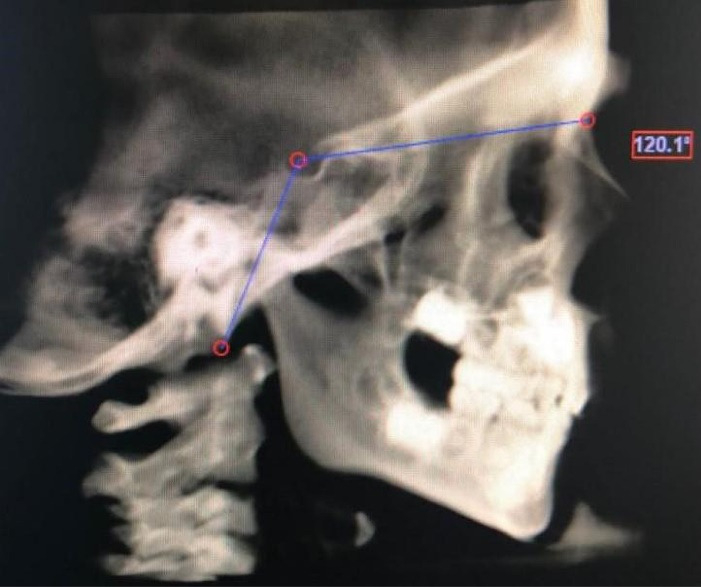
Measurement of BaSN angle

**Figure 2:**
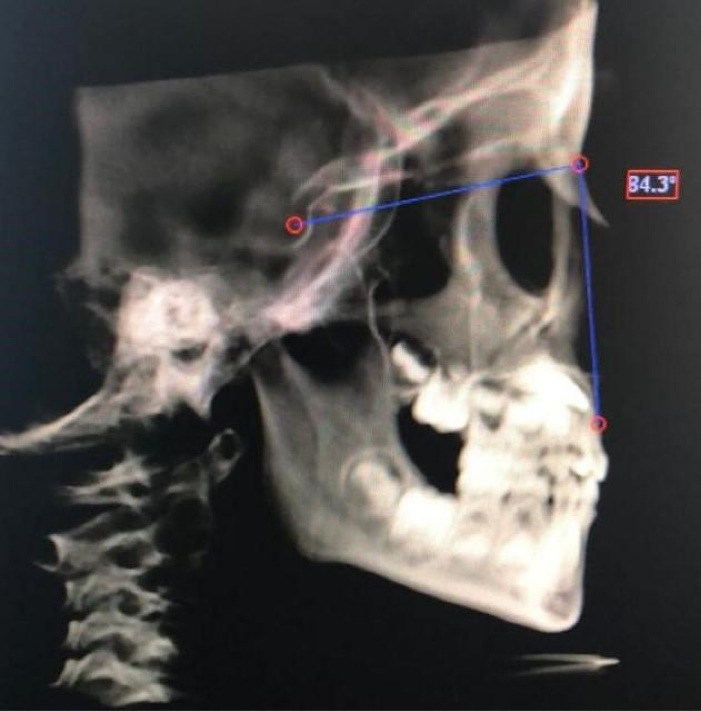
Measurement of SNA angle.

**Figure 3:**
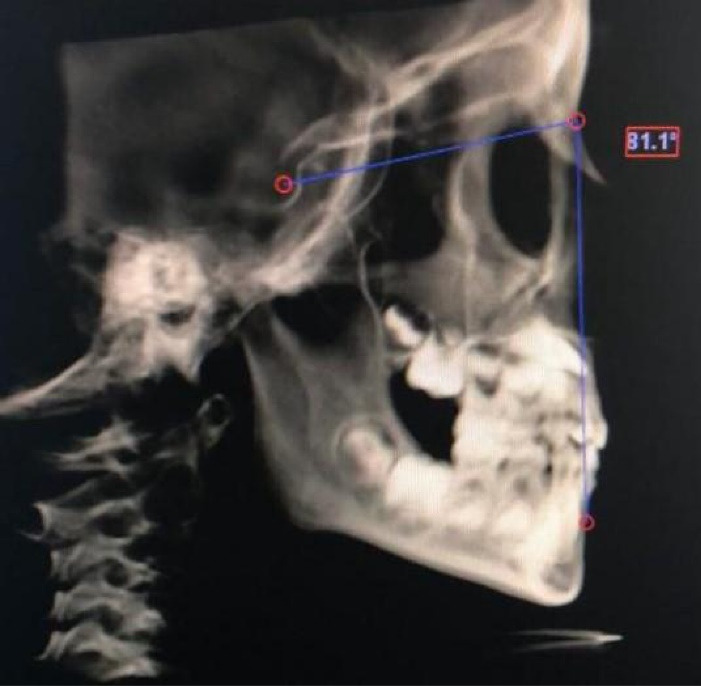
Measurement of SNB angle.

**Figure 4:**
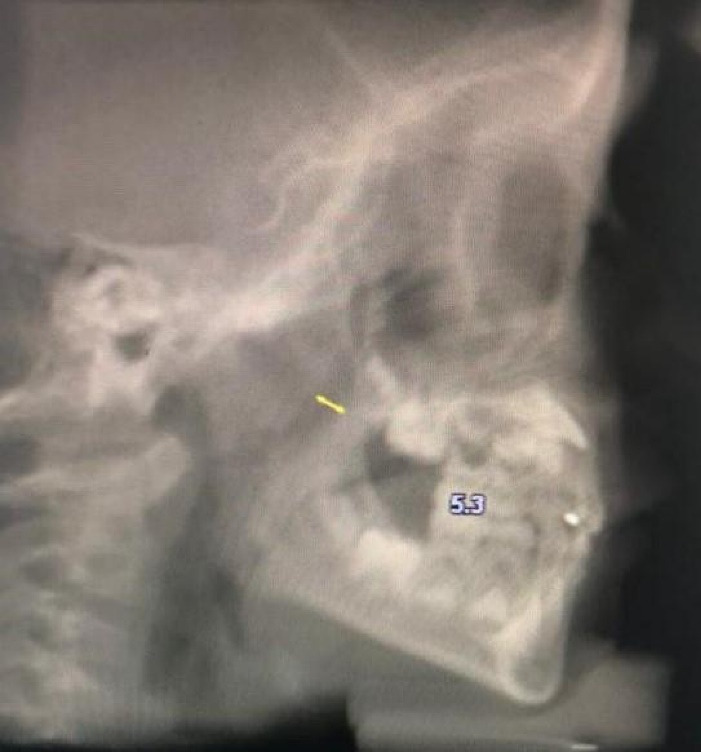
Measurement of PNS-AD1 distance.

Table 1 presents the cephalometric and other variables evaluated in this study. Figures 1-4 show some cephalometric measurements.

**Table 1:** Cephalometric and other variables evaluated in this study and their measurement methods

**Variables**	**Measurement method**
SNA	The angle created between sella, nasion, and A points
SNB	The angle created between sella, nasion, and B points
ANB	Difference of SNA and SNB angles
SN-MP	The angle between SN line and mandibular plane
PP-MP	The angle between the palatal plane and mandibular plane
BaSN	The angle created between basion, sella, and nasion points
PNS-AD1	Shortest distance between PNS and Adenoid tissue
PNS-AD2	Distance between PNS and adenoid tissue on the line perpendicular to BaS from PNS

###  Statistical analysis

 The results of this study were reported by descriptive statistics (mean ± SD, frequencies, and percentages). Kolmogorov-Smirnov test showed normal distribution of quantitative variables (Table 2); therefore, independent samples *t* test was used to compare data between the groups. Data analysis was performed using SPSS 17, and *P* < 0.05 was considered statistically significant.

**Table 2:** Normality of data distribution using Kolmogorov-Smirnov test

**Variable**	**Test statistic**	***P*** ** value**
UA volume (mm^3^)	1.064	0.207
UA area (mm^2^)	0.975	0.298
UA length (mm)	0.629	0.824
UA Ant-Post dimension (mm)	0.733	0.656
UA width (mm)	0.6	0.865
SNA (degrees)	2.150	0.228
SNB (degrees)	0.619	0.838
ANB (degrees)	0.697	0.716
SN-MP(degrees)	0.687	0.732
PP-MP (degrees)	0.855	0.457
IMPA (degrees)	1.506	0. 212
BaSN (degrees)	0.587	0.881
PNS-AD1 (mm)	0.561	0.911
PNA-AD2 (mm)	0.685	0.735

## Results

 The OSA group consisted of 12 female (48%) and 13 male (52%) patients (mean age = 9.25 ± 1.08 years). Twelve females (48%) and 13 males (52%) were included in the control group (mean age = 9 ± 2.56 years).

Table 3 presents the means ± SD of all the measured variables in each group and compares variables between the two groups using an independent *t* test.

**Table 3:** Means ± SD of all the variables in each group and comparison of the variables between OSA and control group using independent *t* test

**Variable**	**OSA group**	**Control group**	***P *** **value**
UA volume (mm^3^)	3670.34 ± 2161.81	7456.37 ± 299.56	0.606
UA area (mm^2^)	2071.64 ± 2161.81	2686.82 ± 1592.98	0.547
UA length (mm)	30.67 ± 2.07	37.18 ± 3.64	0.706
UA Ant-Post dimension (mm)	6.94 ± 3.57	7.59 ± 0.99	0.028
UA width (mm)	16.11 ± 1.99	23.33 ± 2.49	0.037
SNA (degrees)	82.63 ± 3.06	74.66 ± 3.67	0.366
SNB (degrees)	76.76 ± 7.05	73.46 ± 2.24	0.012
ANB (degrees)	5.86 ± 4.01	2.86 ± 1.60	0.114
SN-MP(degrees)	40.15 ± 4.88	33.33 ± 4	0.902
PP-MP (degrees)	23.93 ± 6.91	12.7 ± 3.85	0.801
BaSN (degrees)	125.7 ± 5.02	142.46 ± 5.51	0.041
PNS-AD1 (mm)	5.6 ± 0.3	8.23 ± 1.12	0.913
PNA-AD2 (mm)	30.13 ± 4.35	37.83 ± 3.09	0.015

 According to the results, UA area, volume, and length were smaller in the OSA group compared to the control group, but the difference was not statistically significant (*P* = 0.547, 0.606, and 0.706, respectively).

 The width and the anteroposterior dimension of UA were significantly lower in the OSA group compared to the control group (*P* = 0.028 and 0.037, respectively).

 There was no statistically significant difference between groups regarding SNA (P = 0.366), ANB (*P* = 0.114), SN-MP (*P* = 0.902), PP-MP (P = 0.801), and PNS-AD1 (*P* = 0.913).

 SNB values were higher in the OSA group (*P* = 0.012); on the other hand, BaSN angle and PNS-AD2 values were lower in the OSA group than healthy participants (*P* = 0.041 and 0.015, respectively).

## Discussion

 There is a gap in studies conducted to evaluate factors related to OSA in children, while most studies are based on the adult population. The study of growth patterns is fundamental in developmental disorders such as OSA. It is noteworthy to mention that finding 50 human subjects who meet inclusion criteria in the specified age range was a difficult task that we managed to accomplish. On the other hand, upper airway structures undergo maturational changes from childhood to adult life.^18^ Therefore, evaluating UA in OSA patients during different age ranges is of great value. In this study, UA investigation was performed on 8‒12-year-old children for the first time, whose UAs are changing due to continuous growth; also, respiratory problems can cause important health issues.^4^ Understanding the morphological etiology of OSA in growing children might lead to more precise and effective treatment approaches.

 Upper airway obstruction can occur in one or several areas from nostrils to the larynx, which needs to be precisely identified to increase treatment effectiveness and improve patients’ quality of life.^19^ Three-dimensional imaging techniques such as magnetic resonance imaging (MRI), computed tomography (CT), and CBCT can be employed for the 3D evaluation and analysis of organs; therefore, they might help detect areas of obstruction or decreased dimensions of UA as well.^20^ This study used CBCT to evaluate UA dimensions and other cephalometric measurements, which provide a 3D display of head and neck hard and soft tissues with lower x-ray exposure to the patient compared to CT imaging.^21^

###  UA dimensions

 It seems logical that some UA dimensions in OSA patients differ from those in healthy children; in this study, we evaluated the width, area, length, anteroposterior dimension, and volume of UA in OSA and healthy children. The results revealed that UA width and UA anteroposterior dimensions were significantly lower in the OSA group, with no significant difference in UA area, length, and volume between healthy and OSA children. It seems reasonable that lower UA area, volume, and other UA dimensions can lead to resistance to airflow, making the person susceptible to OSA.

 Enciso et al^1^ reported that UA width in CBCT measurements was significantly lower in the OSA group than in the healthy group. In a study by Buchanan et al,^22^ UA width was significantly lower in the OSA group, confirming the findings of the current study. Volume and area were significantly lower in the OSA group, consistent with the current study results. However, these findings were not statistically significant in our study, which might be related to different ethnic groups compared to our study. Also, in Buchanan and colleagues’ study, UA was longer in the OSA group, which contrasts our results. The reason might be the use of different points for defining UA length (hard palate to epiglottis) compared to our study. However, a study by Neelapu et al^23^ was consistent with our study regarding longer UA in OSA patients due to lower hyoid position.

 MRI assessment of UA dimensions by Arens et al^24^ showed that the UA area was significantly lower in children with OSA than in the control group, indicating the same but non-significant difference in our study. These findings can be justified by considering the higher sensitivity of MRI for soft tissue evaluation compared to CBCT.

 This study also evaluated some cephalometric measurements:

- SNA, SNB, ANB as indicators of jaws position and relationship
- BaSN angle to show the amount of cranial base flexion
- SN/MP and SN/PP as indicators of vertical growth pattern
- PNS to AD1 and AD2 distances to represent adenoid tissue projection in the pharyngeal space

Although SNA and ANB values showed no statistically significant differences between the two groups, the SNB angle was significantly larger in the OSA group. Therefore, there was no significant difference in maxillary anteroposterior position between the two groups; however, the mandible was significantly more anteriorly positioned in the OSA group according to SNB measures, contrasting some previous studies like Ryu et al,^25^ who claimed that mandibular retrognathism had a positive correlation with OSA severity. However, some other studies failed to find any differences in mandibular position between OSA and control groups.^26^ Considering the age limit of this study’s samples in whom the mandible is still actively growing, this distinct result is significant, suggesting that mandibular retrognathism might not be a risk factor for OSA in children. Similarly, the results of ANB comparisons between the two groups in this study showed that skeletal anteroposterior relationships might play no significant role in OSA development in children.

 SN/MP and PP/MP angles were higher in healthy samples, with no statistically significant differences. Therefore, it can be concluded that the vertical dimension might not be responsible for OSA in children.

 BaSN angle was significantly lower in the OSA group, consistent with studies that showed a positive correlation between cranial base flexion and anteroposterior airway dimensions that might influence OSA development.^23,27^

 Similar to previous studies, a significantly smaller distance between PNS and AD2 and a non-significant, smaller distance between PNS and AD2 in the current study demonstrate the possible role of adenoid tissue enlargement in OSA development in children.^28,29^

## Conclusion

 The results of this study demonstrated that

 Reduced UA dimensions might play an important role in OSA development in children; however, most of the skeletal variables, such as the anteroposterior relationship of jaws and jaw rotations, were not significantly different between children with OSA and the control group.

 There was significantly more flexion of the cranial base in the OSA group, resulting in a narrower UA in these children.

 Adenoid tissues were larger in children with OSA compared to healthy children.

## Acknowledgments

 We would like to acknowledge the staff of the Department of the Oral and Maxillofacial Radiology, Faculty of Dentistry, Tabriz University of Medical Sciences, who warmly helped us in performing the study.

## Authors’ Contributions

 FE and AHS planned the study. AHS, MS, and AN performed the study. PN and AHS performed the literature review. PN drafted the manuscript, and FE carried out the statistical analyses and interpretation of data. AHS critically revised the manuscript for intellectual content. All the authors have read and approved the final manuscript.

## Funding

 This study was not supported financially by any organization.

## Ethics approval

 This study was approved ethically by the Ethics Committee of Tabriz University of Medical Sciences: IR.TBZMED.REC.1397.150.

## Competing interests

 The authors report no conflict of interest in this study.
